# Atomistic modelling of scattering data in the Collaborative Computational Project for Small Angle Scattering (CCP-SAS)^1^

**DOI:** 10.1107/S160057671601517X

**Published:** 2016-10-14

**Authors:** Stephen J. Perkins, David W. Wright, Hailiang Zhang, Emre H. Brookes, Jianhan Chen, Thomas C. Irving, Susan Krueger, David J. Barlow, Karen J. Edler, David J. Scott, Nicholas J. Terrill, Stephen M. King, Paul D. Butler, Joseph E. Curtis

**Affiliations:** a Department of Structural and Molecular Biology, University College London, Darwin Building, Gower Street, London WC1E 6BT, UK; b Center for Neutron Research, National Institute of Standards and Technology, Gaithersburg, MD 20899-8562, USA; c Department of Biochemistry, University of Texas Health Science Center at San Antonio, San Antonio, TX 78229-3900, USA; d Department of Biochemistry and Molecular Biophysics, Kansas State University, Manhattan, KS 66506, USA; e Department of Biology, Illinois Institute of Technology, 3101 S. Dearborn, Chicago, IL 60616, USA; f Pharmacy Department, Franklin-Wilkins Building, King’s College London, 150 Stamford Street, London SE1 9NH, UK; g Department of Chemistry, University of Bath, Claverton Down, Bath BA2 7AY, UK; h School of Biosciences, University of Nottingham, Sutton Bonington Campus, Leicestershire LE12 5RD, UK; i Research Complex at Harwell, STFC Rutherford Appleton Laboratory, Harwell Campus, Didcot, Oxfordshire OX11 0FA, UK; j ISIS Facility, STFC Rutherford Appleton Laboratory, Harwell Campus, Didcot, Oxfordshire OX11 0QX, UK; k Diamond Light Source Ltd, Diamond House, Harwell Science and Innovation Campus, Chilton, Didcot, Oxfordshire OX11 0DE, UK; l Department of Chemistry, The University of Tennessee, Knoxville, TN 37996-1600, USA

**Keywords:** molecular dynamics (MD), molecular modelling, scattering curve fits, small-angle-neutron scattering (SANS), small-angle-X-ray scattering (SAXS)

## Abstract

The capabilities of current computer simulations provide a unique opportunity to model small-angle scattering (SAS) data at the atomistic level, and to include other structural constraints ranging from molecular and atomistic energetics to crystallography, electron microscopy and NMR. This extends the capabilities of solution scattering and provides deeper insights into the physics and chemistry of the systems studied. Realizing this potential, however, requires integrating the experimental data with a new generation of modelling software. To achieve this, the CCP-SAS collaboration (http://www.ccpsas.org/) is developing open-source, high-throughput and user-friendly software for the atomistic and coarse-grained molecular modelling of scattering data. Robust state-of-the-art molecular simulation engines and molecular dynamics and Monte Carlo force fields provide constraints to the solution structure inferred from the small-angle scattering data, which incorporates the known physical chemistry of the system. The implementation of this software suite involves a tiered approach in which *GenApp* provides the deployment infrastructure for running applications on both standard and high-performance computing hardware, and *SASSIE* provides a workflow framework into which modules can be plugged to prepare structures, carry out simulations, calculate theoretical scattering data and compare results with experimental data. *GenApp* produces the accessible web-based front end termed *SASSIE-web*, and *GenApp* and *SASSIE* also make community SAS codes available. Applications are illustrated by case studies: (i) inter-domain flexibility in two- to six-domain proteins as exemplified by HIV-1 Gag, MASP and ubiquitin; (ii) the hinge conformation in human IgG2 and IgA1 antibodies; (iii) the complex formed between a hexameric protein Hfq and mRNA; and (iv) synthetic ‘bottlebrush’ polymers.

## Abstract 2

The CCP-SAS project is currently developing software for the atomistic and coarse-grained molecular modelling of X-ray and neutron small-angle scattering data. Its computational framework is described, alongside applications in biology and soft matter.

## 1. Introduction   

Small-angle X-ray scattering (SAXS) and neutron scattering (SANS) are diffraction techniques for investigating a broad range of science. Here, we are particularly interested in their use in investigations of the structural properties of biomaterials, including proteins, nucleic acids and polysaccharides, and soft condensed matter systems, including synthetic polymers, micelles and liquid crystals (Perkins *et al.*, 2008 ▸, 2011 ▸; Svergun *et al.*, 2013 ▸; Higgins & Benoit, 1996 ▸; Gabrys, 2000 ▸). Like macromolecular crystallography, SAXS and SANS experiments have benefited hugely from major infrastructural investment in high-brilliance multiuser X-ray synchrotrons and neutron sources during the past two decades. Instrumental improvements that include higher beam intensities, the automation of data collection and more sensitive detector technologies have vastly increased both the throughput of samples and the quality of SAXS and SANS data (Hura *et al.*, 2009 ▸; Pernot *et al.*, 2013 ▸; Round *et al.*, 2015 ▸; Heenan *et al.*, 2011 ▸; Dewhurst *et al.*, 2016 ▸).

As these are low-resolution techniques, detailed analysis of small-angle scattering (SAS) data requires the use of as much *a priori* knowledge about the system as possible. Thus SAS analysis often begins with semi-quantitative techniques such as radius of gyration *R*_G_ fits or pair density distribution *P*(*r*) functions (Feigin & Svergun, 1987 ▸). The traditional and most straightforward approach to quantitative structural analysis of SAS data makes assumptions about the class of structure under investigation. The SAS from that object (the form factors and/or structure factors) is then calculated analytically using the parameters of the class (*e.g.* radius and density/composition for a sphere) as the unknown pieces of information to extract from the data. A large number of form factors have been derived and new ones continue to be published, but such analytical solutions are restricted to shape classes with sufficiently simple symmetry. In order to address the need to fit data from objects with little or no symmetry (ubiquitous in biomaterials such as proteins), non-atomistic real space and *ab initio* approaches were developed, most notably in the *ATSAS* suite of programs from EMBL Hamburg in which shape envelopes from spherical harmonics or assemblies of small spheres are used to fit experimental SAS data (Petoukhov *et al.*, 2012 ▸; https://www.embl-hamburg.de/biosaxs/software.html). However, other than the minimal assumptions built into the shape optimization algorithm, no *a priori* information is used other than that gained from long experience and expert knowledge, often limiting the level of detail accessible. Increases in computer power and sophistication have led a number of groups to use known atomic coordinates (obtained for example from a crystal structure) to create atomistic models of macromolecules to fit SAXS and SANS data, including the use of automated fit procedures; other groups add further *a priori* information by including some type of energetic constraint (reviewed by Perkins *et al.*, 2008 ▸, 2009 ▸, 2011 ▸; Lipfert & Doniach, 2007 ▸; Putnam *et al.*, 2007 ▸; Rambo & Tainer, 2013 ▸). Indeed, despite the inherently low resolution of SAS and the loss of information due to orientational averaging, atomistic level structural models can be derived, with the bonding and energetic constraints they impose, when advanced molecular simulation is used to derive physically plausible configurations. These models are consistent not only with the scattering data but with at least some of the known physical chemistry of the systems, and provide functional insights for tests by further experimentation. An extensive but not exhaustive list of SAS data fitting packages, organized into categories, is available for download on the canSAS-maintained SAS portal at http://smallangle.org/content/Software.

**Figure 1:**
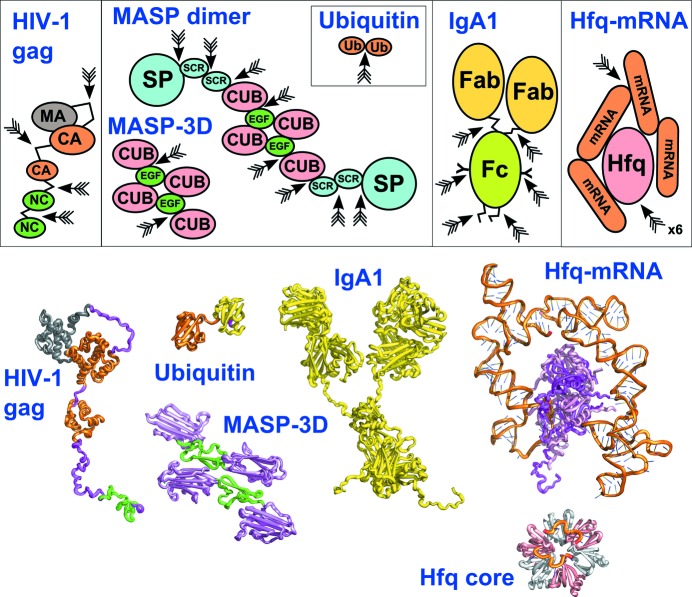
Upper panel: Schematic domain structures of six *SASSIE*-modelled macromolecules. The domains are drawn approximately to scale according to their molecular structures. The major linkers varied in *SASSIE* searches are denoted by arrows (Table 1); some simplification has been made. The domain names are as follows: HIV-1 Gag: MA, matrix; CA, capsid; NC, nucleocapsid. MASP: CUB, C1r/C1s, Uefg and bone morphogenetic protein-1; EGF, epidermal growth factor; SCR, short complement regulator; SP, serine protease. Ub, ubiquitin. IgG2 and IgA1: Fab, fragment antigen binding; Fc, fragment crystallizable. Lower panel: Molecular structures for these six macromolecules, all drawn to the same scale in *PYMOL* (Schrodinger LLC). The best-fit structures are to be described as ensembles of structures and not as the single structures as shown. The domain colours follow those in the upper panel. That for HIV-1 Gag is taken from the starting model for the simulations, where the MA, CA and NC domains are taken from crystal or NMR structures, and the p2 domain is not shown (Table 1 ▸). That for MASP-3D is taken from the crystal structure of MASP-1 and was the starting model used to initiate the fitting. That for the K27-ubiquitin dimer is taken from the isopeptide dimer formed through Lys27 (distal Ub, orange; proximal Ub, yellow; K27, magenta). That for IgA1 is the final model from the *SASSIE* fits, but not showing the glycan chains (Fig. 4). That for the Hfq–mRNA complex is the input file used in the Complex MC tutorial; under this is the starting crystal structure of the Hfq core protein bound with two heptamer nucleotide chains (PDB code 4ht8). The mRNA chains are shown in orange.

The use of atomistic modelling to inform SAS analysis dates back several decades. For example, the first atomistic modelling, based on crystal structures but constrained by scattering curves, of antibody structures for the three Fab and Fc fragments (Fig. 1 ▸) showed that only a limited number of fragment conformations would fit the X-ray or neutron scattering curves (Perkins *et al.*, 1991 ▸; Mayans *et al.*, 1995 ▸). The first automation of atomistic scattering modelling was reported for the X-ray and neutron scattering fits for human IgA1 antibody using the *SCT* and *SCTPL* software tools with a commercial molecular dynamics (MD) package (Boehm *et al.*, 1999 ▸). The hinge (or linkers) between crystal structures for the Fab and Fc regions were moved to explore relevant configuration space using MD to generate 12 000 trial full structures, of which only 102 gave good X-ray and neutron fits. This resulted in the first atomistic solution scattering structure to be deposited in the Protein Data Bank (PDB code 1iga). Since that time, *SCT* and *SCTPL* modelling has resulted in 77 such structures (Wright & Perkins, 2015 ▸), including the antibody classes of adaptive immunity (Fig. 1 ▸), the complement proteins of innate immunity with as many as 30 small domains, and linear anionic oligosaccharides containing up to 36 carbohydrate rings (Perkins *et al.*, 2008 ▸). When comparisons were made, these structures compared well with those from other methods, such as protein crystallography (Perkins *et al.*, 2008 ▸, 2011 ▸). During the past decade, several other groups have also pursued such modelling approaches (Whitten *et al.*, 2008 ▸; Pelikan *et al.*, 2009 ▸; Yang *et al.*, 2009 ▸, 2010 ▸; Schneidman-Duhovny *et al.*, 2010 ▸, 2016 ▸; Poitevin *et al.*, 2011 ▸; Różycki *et al.*, 2011 ▸; Evrard *et al.*, 2011 ▸; Ihms & Foster, 2015 ▸; Chen & Hub, 2015 ▸; Jimenez-Garcia *et al.*, 2015 ▸; Knight & Hub, 2015 ▸).

Despite these efforts, there has still been no significant shift toward atomistic or coarse grained modelling for SAXS and SANS. Further, there have been very few efforts to keep pace with rapidly evolving simulation methods through increases in computer power, coupled with new realistic force fields and robust sophisticated simulation engines. To begin addressing this issue, a modelling framework, termed *SASSIE*, emerged in 2004 at the NIST Center for Neutron Research (Datta *et al.*, 2007 ▸; https://sassie-web.chem.utk.edu/sassie2/). *SASSIE* was developed to provide a general modular framework that enabled modern simulation methods to be applied to model scattering data using physical constraints (Curtis *et al.*, 2012 ▸). It is important to understand that *SASSIE* is a framework built on a plugin architecture. It is meant to be agnostic to the particular MD engine, force field, type of material or approach to solving the molecular structure. Any limitations in that regard come strictly from what modules are available. In fact, many if not all of the atomistic efforts developed so far could in principle be wrapped into modules that take advantage of the integrated *SASSIE* workflow. The other new element in *SASSIE* was the incorporation of Monte Carlo (MC) simulation methods to create ensembles of biomolecular structures by sampling user-selected backbone dihedral angles to model experimental X-ray and neutron data. Without this advance, the generation of atomistic structures using modern force-field-based simulations could take months or even be inaccessible. Because robust, force-field-based structures are used throughout the *SASSIE* workflow, most modern simulation packages can be used to model SAS data in detail. Through the generation of robust and complete physics models using best-practice simulation methods, the resulting ensemble of structures can be fitted to SAXS and SANS data within the integrated workflow.

Despite the advances through the development of *SASSIE*, four key issues remain to make various SAS modelling problems more tractable. Firstly, regarding accessibility, because the *SASSIE* framework supports an array of plugin modules, the end user installation of the software could be frustrating and require a level of technical knowledge usually lacking in a typical non-expert SAS end user. New versions can quickly overwhelm the users and the small development team. Secondly, it became quickly evident that the largest barrier for the non-simulation end users was the need for expert knowledge to prepare the correct starting trial structural models and the associated protein structure files. The general difficulty for biological non-experts is creating a well constructed starting structure, while that for soft matter non-experts is that the appropriate force fields may not be available. Thirdly, the increasing complexity of systems of interest is leading to an increasing need to run these simulations on high-performance computing (HPC) resources, something outside the skill set of most SAS users. Fourthly, it has become clear that developing and maintaining the *SASSIE* framework, while also wrapping or developing the possible and desired packages and tools as new modules plugged into the *SASSIE* framework, is beyond the ability of a single small group to manage. This requires a larger community effort.

The 17 Collaborative Computational Projects (CCPs) in the UK (as of August 2016; http://www.ccp.ac.uk/) provide a software infrastructure to build individual research projects and to maintain and distribute code libraries. In order to reveal how atomic level molecular structures in biological or soft matter systems account for experimental scattering data, the Collaborative Computational Project for Small Angle Scattering (CCP-SAS), jointly funded by the EPSRC research council in the UK and the National Science Foundation in the USA, was created in 2012 to address these issues of access and long-term sustainability. The specific initial goals of the consortium were to (i) significantly lower the barrier for bench scientists to access the power of high-end state-of-the-art molecular modelling and computational chemistry tools; (ii) provide a user-friendly software environment that integrates SAS data with those tools for purposes of structural refinement, further informed by data from complementary techniques such as analytical ultracentrifugation, electron microscopy or NMR; and (iii) build a long-term development and maintenance support structure through community development and engagement with large-scale SAS user facilities as well as other CCPs. Here, we provide an overview of the CCP-SAS project, focusing heavily on its current core activities. These comprise the development of a new *GenApp* infrastructure for deployment of computational code, the ongoing development of the *SASSIE* framework and its implementation as *SASSIE-web* powered by the new *GenApp* package to provide a web front end and HPC back end, and the ongoing development of the workflow of modules required to address molecular simulations, scattering calculators and the analyses of their output. For some soft matter systems, the extension of *SASSIE* to coarse-grained and hybrid methods (mixing shapes with atomistic structures) will be important. To illustrate some representative atomistic modelling workflows, we summarize applications of *SASSIE-web* to a broad range of systems in biology and soft matter (Fig. 1 ▸) (Datta *et al.*, 2007 ▸; Nan *et al.*, 2017 ▸, unpublished work; Castañeda, Chaturvedi *et al.*, 2016 ▸; Castañeda, Dixon *et al.*, 2016 ▸; Clark *et al.*, 2013 ▸; Hui *et al.*, 2015 ▸; Peng *et al.*, 2014 ▸; Zhang *et al.*, 2014 ▸).

## 2. Methods: the CCP-SAS software portfolio   

### 2.1. Summary   

The initial goal of CCP-SAS is to provide an open-source cloud-based software environment that not only makes clear how the modelling fit analyses were performed, and permits experimental teams to understand complex chemical interactions and structural organizations, but is flexible enough to incorporate additional different experimental constraints into the modelling workflow. The CCP-SAS project also aims to provide documentation and training, and ultimately to foster a sustainable community of users. This user base includes experimental research groups, software developers and instrument scientists at multiuser scattering facilities.

Current CCP-SAS activities include these nine tasks: development of the *GenApp* infrastructure and *SASSIE* framework; deployment of *SASSIE* as *SASSIE-web* to the community; wrapping existing code and developing new code as new modules for *SASSIE*; developing new methods for eventual incorporation as new modules in *SASSIE*; working with members of the SAS community to implement their relevant methods and codes into the *SASSIE* framework; providing help and guidance to members of the SAS community to wrap their standalone codes using *GenApp* for separate web deployment outside of *SASSIE-web*; where feasible and reasonable, hosting such separate web applications on the CCP-SAS cluster for the benefit of the community; running tutorials and workshops; and working to engage various community stakeholders.

Two core principles of CCP-SAS are to use both existing and open-source software as much as possible. If a critical non-open-source component is needed, it can be incorporated, but then an alternative open-source solution is identified to replace this as quickly as possible. This policy accommodates the drive for open-source software for proper validation and transparency increasingly requested by funding bodies and helps engage community support. Thus all CCP-SAS software, including *SASSIE* and *GenApp*, is freely available and open source. While one closed-source package currently remains (August 2016), this will be removed as soon as the alternative modules are validated.

### 2.2. The *GenApp* deployment infrastructure   

**Figure 2:**
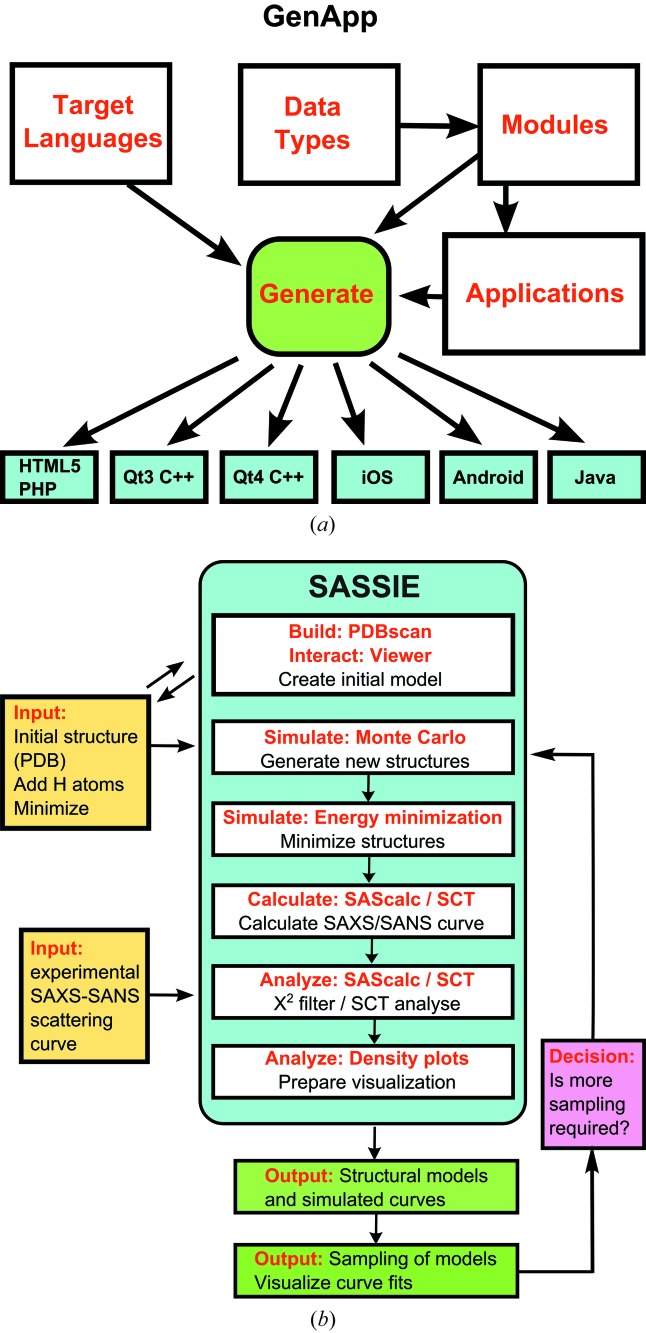
The *GenApp* and *SASSIE* infrastructures. (*a*) The use of *GenApp* to generate applications. The generator (green box) reads application definitions, module definitions and chosen target language information to assemble the application instances. Examples of target languages are shown in the cyan boxes (adapted from Brookes *et al.*, 2015 ▸). In application to *SASSIE*, *GenApp* is able to take any set of executables (created using any set of programming languages) compatible with a certain platform (*e.g.* Windows or Linux) and present them together in the single web interface that is shown in Fig. 3(*a*). (*b*) In the *SASSIE* workflow, the schematic relationships between the *SASSIE* framework and five of the six main modules within *SASSIE* are shown within the cyan box. These modules are assembled using the *GenApp* deployment infrastructure. The two inputs for *SASSIE* are shown in yellow boxes. The two outputs from *SASSIE* are shown in green boxes. At this point, a decision is required in terms of whether the modelling is completed (red box).

The *GenApp* infrastructure was developed to simplify the deployment of CCP-SAS software (Brookes *et al.*, 2015 ▸). Common issues addressed by *GenApp* include easing the deployment of a workflow of modules, support for legacy codes and the reduction of dependencies on dedicated software teams. This is achieved by enabling the generation of web-based and standalone graphical user interface (GUI) applications over the same underlying executable software while providing transparent access to back end computational resources and connections to high-performance computing gateways (Fig. 2 ▸ *a*). Long-term sustainability questions are addressed by decoupling the GUI and back end interfaces from the core computational codes, such as the *SASSIE* suite being developed. *GenApp* is thus the core technology to address the accessibility issues as well as the long-term sustainability issues.

In *GenApp*, an application is defined as a collection of executable modules which are presented through a common user interface (Fig. 2 ▸ *a*). This provides a powerful paradigm to combine both existing and new codes in order to perform novel workflows or develop different types of modelling applications. The addition of a module in *GenApp* is simple, and only requires the writing of a short JSON wrapper (a module) to detail the input and output, and the editing of two JSON files, one to specify where the module should appear in the applications menu system, and the other to specify how the application itself is to be presented. The modules themselves can be written in any supported language, independent of the choice of the target GUI implementation. Separating the scientific code from the GUI not only facilitates the linking of component modules into larger workflows and applications, but also reduces the burden in supporting legacy codes. *GenApp* also facilitates the creation of applications as web servers or gateways. This includes remote file management and the execution and management of lengthy non-interactive jobs. The latter capability, provided through integration with Apache Airavata (https://airavata.apache.org/), allows *Gen­App* applications to harness a range of high-performance computing resources including local clusters, supercomputers, national grids, and academic and commercial clouds. We anticipate that *GenApp* will be useful to generate a wide range of scientific applications beyond the scope covered by CCP-SAS.

*GenApp* was designed to be generic, and thus its power is available to any developers seeking to take advantage of the ease of deployment and transparent access to high-end computing resources it offers. *GenApp* modules can be part of a module developer’s standalone application or hosted in CCP-SAS computer resources as a public web-based science gateway. *GenApp* web applications can in principle be deployed on any cloud resource, and instances have been tested on XSEDE (https://www.xsede.org/) and AWS (https://aws.amazon.com/). The developed *GenApp* module can be added to our open repository. Currently the project is working with SAS developers including *WillItFit* (Pedersen *et al.*, 2013 ▸) and *QuaFit* (Spinozzi & Beltramini, 2012 ▸). Both packages are deployed for alpha testing as web applications hosted on our CCP-SAS resources. Interested parties are invited to send an email message to genapp-devel@biochem.uthscsa.edu.

### 2.3. The *SASSIE-web* workflow   

**Figure 3:**
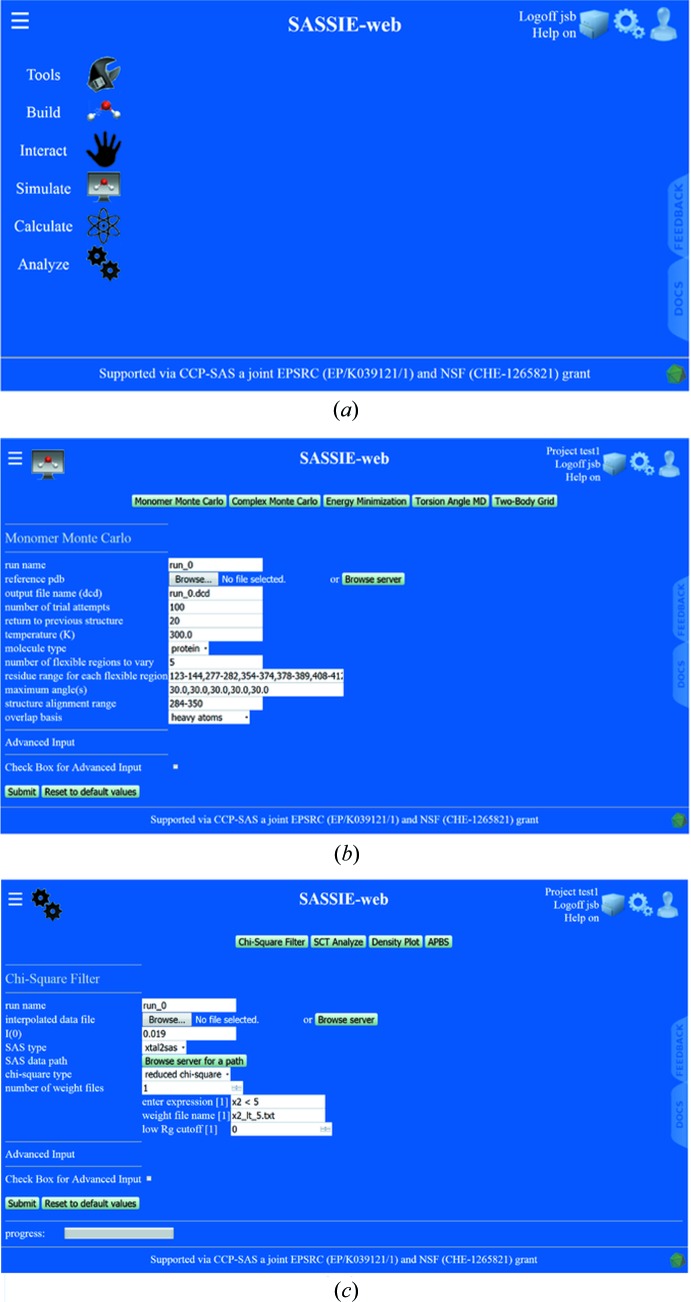
The *SASSIE-web* user interface. (*a*) The home page at https://sassie-web.chem.utk.edu/sassie2/. The six main modules of *SASSIE* are shown to the left. (*b*) The input screen to set up a Monomer MC simulation from the Simulate module is shown. (*c*) The χ^2^ filter input screen from the Analyze module is shown.

The aim of *SASSIE-web* is to allow experimentalists (including novice users) to construct their own modelling workflows from a set of simulation and analysis modules, then run them transparently on centrally maintained back end resources for scattering curve comparisons, from nothing more than a web browser (https://sassie-web.chem.utk.edu/sassie2/) (Fig. 3 ▸). The provision of a web interface avoids the need for users to install and maintain large complex software on their own machines, and facilitates the provision of a high-performance computing back end to accelerate the computationally expensive steps of the modelling process. The *SASSIE-web* menu organizes the workflow in terms of six sets of modules (Fig. 3 ▸ *a*): (i) Tools, which includes utilities to predict scattering length densities, interpolate experimental scattering data files when required, and extract or merge macromolecular structures; (ii) Build, which includes utilities to check PDB-formatted coordinate files; (iii) Interact, which provides a molecular viewer to present an interactive display of a specified structure using *JSMol* (Hanson *et al.*, 2013 ▸); (iv) Simulate, which provides the modules that create the representative ensemble of trial structures for test against the data (Fig. 3 ▸ *b*); (v) Calculate, which provides a range of scattering curve calculators; and (vi) Analyze, which determines the goodness of fit between the simulated and experimental scattering curves in order to identify the best-fit scattering structure (Fig. 3 ▸ *c*) and provide visualizations to display the trial structures and the best-fit subset of these as envelopes.

The modular design of *SASSIE* not only gives the user the freedom to employ any combination of existing modules but also allows them to plug in new modules, and import coordinate models generated with other packages at any stage of the workflow. This modular nature of *SASSIE*, combined with the ease of deployment and end user accessibility, makes *SASSIE-web* an attractive option for SAS computational groups wishing to contribute their codes. For example, the *Capriqorn* software to calculate scattering curves from molecular simulations with explicit water models is being integrated into the *SASSIE* framework (Köfinger & Hummer, 2013 ▸). Interested parties are invited to email joseph.curtis@nist.gov.

### 2.4. Validation of starting coordinate models using *PDB-scan*   

The *SASSIE* workflow is summarized in Fig. 2 ▸(*b*). The modelling of scattering data is crucially dependent on correct starting atomistic models. Even though over 121 000 structures (August 2016) are available in the PDB, it does not follow that these are ready for molecular simulation and scattering curve calculations. Common problems are gaps in the protein structure caused through disorder (especially at surface loops), errors in the amino acid sequence, missing structures such as incomplete glycan chains or N-terminal or C-terminal sequences, and missing or misnamed atoms (*e.g.* hydrogen atoms, carbon atoms and disulphide bridges). If the structure of interest is not available in the PDB, standard homology (comparative) modelling techniques which are necessarily outside *SASSIE* (such as *MODELLER*; Šali & Blundell, 1993 ▸) can be used to generate the input structure from the most closely related protein structure in the PDB. In this process, the amino acid sequence will need to be replaced by the sequence of interest. *PDB-scan* assesses whether a PDB file is ready for a scattering curve simulation, and where possible provides files enabling *CHARMM* force-field parameterization (MacKerell *et al.*, 1998 ▸; Best *et al.*, 2012 ▸). Scans provide information on missing atoms and residues and those not covered as standard by the *CHARMM* force field. *PDB-scan* also reports on whether symmetry information present in the PDB header can be used to create a dimer or higher-order oligomer that is the actual biological unit to be modelled. Suitable coordinate files can be derived from the output that are ready to be used by a wide range of simulation and modelling software packages, including those focused on soft matter systems. To complement the capabilities of *PDB-scan*, a new module termed *PDB-Rx* is in preparation to correct mistakes discovered by *PDB-scan* (Wright *et al.*, 2016 ▸).

### 2.5. Generation of molecular ensembles   

In *SASSIE* (Fig. 2 ▸ *b*), the key stage of modelling SAXS and SANS data is the generation of ensembles of atomistic structures that sample the configuration space of physically realistic models. Early approaches used various MD or MC methods to vary the appropriate segment in the system of interest (Boehm *et al.*, 1999 ▸; Datta *et al.*, 2007 ▸; Khan *et al.*, 2010 ▸). For biological work, to generate structural models of protein or nucleic acids rapidly, *SASSIE-web* offers dihedral angle MC simulations through the Markov sampling of backbone torsion angles in user-specified regions of the input model (Curtis *et al.*, 2012 ▸). MC simulations can be performed on any PDB structure which contains all atoms in the model. However, in order to make use of the full range of simulation and analysis options in *SASSIE*, it is recommended that the input PDB file is prepared for MD simulation using the *CHARMM* force field. It is not necessary to obtain the *CHARMM* simulation package in order to perform this process. One common approach is to use the structure building tool *PSFGEN* which is distributed openly as a plugin with the *VMD* visualization program or the *NAMD* simulation package (Humphrey *et al.*, 1996 ▸; Phillips *et al.*, 2005 ▸). As an alternative, access to *CHARMM* force-field parameterization is provided by *CHARMM-GUI* (Jo *et al.*, 2008 ▸). The starting input structure must be a complete structure without missing residues (see above) and atom and residue names must be compatible with those defined in the *CHARMM* force field (MacKerell *et al.*, 1998 ▸).

The modelling strategy completely depends on the system of interest. During a typical simulation workflow for a multidomain protein with linkers to be varied between the domains, about three to six linker regions in the starting structure are sampled in the simulation. Depending on the system of interest, around 10 000 to 50 000 structures might be required to sample adequate configuration space for most problems; see Table 1 ▸ for examples. Since steric clashes can easily occur during the simulation, the avoidance of atomic overlap is achieved by specifying an overlap distance cutoff (typically 0.3 nm) and the atom name(s) to which this applies. Other options include the selection of simulated structures to remain within a fixed range of *R*_G_ values and/or satisfy intra- and intermolecular distance constraints. Collections of output structures are stored in the DCD file format used by the *CHARMM*, *NAMD* and *X-PLOR* MD packages (this binary format stores multiple structures much more efficiently than text-based PDB files; Brunger, 1992 ▸). These files can also be visualized in many molecular viewers, such as *VMD* or *Chimera* (Humphrey *et al.*, 1996 ▸; Pettersen *et al.*, 2004 ▸). Presently, two interfaces to MC simulations are provided in the Simulate module, namely Monomer MC and Complex MC. As their names suggest, the former provides a simplified interface focusing on single-chain biosystems, while the latter facilitates the simulation of more complex topologies. A tutorial using the Monomer MC module, based on its original use case of the HIV-1 Gag protein (Fig. 1 ▸) (Datta *et al.*, 2007 ▸), can be found at https://sassie-web.chem.utk.edu/sassie2/docs/sassie-web-quick-start/quick-start.html. While this example covered a workflow for a protein, the other simulation engines such as *NAMD* and *CHARMM* within *SASSIE* enable any molecular system to be simulated, including in particular soft matter systems, something that is the focus of ongoing work.

The outcome of the MC simulations is available to another module that uses energy minimization and MD to sample degrees of freedom not sampled in the MC trajectories from biomolecular models as parameterized in the *CHARMM* force field (Fig. 2 ▸ *b*). *NAMD* (version 2.9) is used as the simulation engine (Phillips *et al.*, 2005 ▸). A reference PDB file name is input, together with the matching starting structure in either PDB or DCD format, and the *CHARMM* topology (PSF) file. The four optional modes of operation are as follows: (i) minimization alone; (ii) minimization followed by MD; (iii) minimization followed by MD leading to a second round of minimization; and (iv) molecular simulation (energy minimization and/or MD) with a user supplied input file. Both the minimization and MD are performed using the generalized Born implicit solvent model. If a DCD file is selected as the input file then the simulations are run on each frame.

Structural models generated by the MC simulations can also be sent to the *Torsional Angle MD* (*TAMD*) module for refinement (Zhang *et al.*, 2016 ▸, unpublished work). *TAMD* samples molecular configurations in torsion angle space, and allows the convenient specification of rigid domains and flexible degrees of freedom consistent with the MC sampling stage (Chen *et al.*, 2005 ▸). For this, the ensemble generated by MC simulations is first sub-sampled to select representative configurations that provide a thorough coverage. Each selected configuration is then used to initiate *TAMD* simulations, which allows refinement of the local structural features and provides improved sampling of conformational degrees of freedom that are not included in the MC moves. Atomistic implicit solvent force fields available in *CHARMM* are used to provide a balance between computational efficiency and efficacy (Chen *et al.*, 2008 ▸). By default, the module currently uses an efficient solvent-accessible surface area (SASA) implicit solvent model (Ferrara *et al.*, 2002 ▸) that can handle proteins, nucleic acids and carbohydrates.

### 2.6. Scattering curve calculators   

Theoretical scattering curves for modelled structures are computed from atomistic positions, such as *via* the Debye equation. As this requires the calculation of distances between every pair of atoms or scattering centres, the computing effort increases with the square of the number of atoms or centres, making this hugely time consuming. These computations become even more computer intensive if the pair distances are convoluted with the scattering length densities of each pair of scattering centres for neutron contrast variation work or for X-ray work with high and low electron densities. Scattering curve calculation can be accelerated by the use of coarse graining of the original atomic structures (resulting in fewer scattering centres), and the use of binning algorithms to reduce the number of distances to be processed. An alternative strategy is to use high-performance computing and graphics processing unit technology to accelerate the computations, both of which have been pursued within CCP-SAS. Several calculators are available in the *SASSIE-web* framework, such as *CRYSOL* and *EM_to_SANS* (Svergun *et al.*, 2012 ▸; Curtis *et al.*, 2012 ▸). Two of the most commonly used are described briefly here.

The *SasCalc* module (Watson & Curtis, 2013 ▸) calculates neutron and X-ray scattering profiles from a user-supplied structure file using an exact all-atom expression for the scattering intensity in which the orientations of the **Q** vectors are taken from a quasi-uniform spherical grid generated by the golden ratio. This ‘golden vector’ method is currently configured to handle atomic trajectory input files (DCD or PDB). Our implementation of the ‘golden vector’ method within *SasCalc* includes corrections for contrast for both X-ray and neutron scattering and harnesses graphical processing units for massively parallel calculations. The use of an atomistic scattering calculator is a vital step towards supporting the full range of soft matter systems beyond biological systems.

The *SCT* module (Wright & Perkins, 2015 ▸) first converts the atomistic structure into a coarse grained sphere model, where each sphere represents about 4–5 atoms, and then employs the Debye equation adapted to small spheres of diameter below the structural resolution of the scattering curves (about 1 nm diameter) to calculate the scattering curve. The simulations utilize single-density spheres. X-ray scattering simulations involve the addition of a hydration monolayer of water molecules in the sphere model creation step, and assume pinhole geometries with X-ray data that are already slit desmeared. Neutron scattering simulations for proteins in heavy water do not require a hydration shell (Perkins, 2001 ▸), but require a smearing correction for wavelength spread and beam divergence in the final scattering curve, as well as a linear buffer background correction for residual incoherent scattering from protons. As part of CCP-SAS, *SCT* was made open source and publicly available and is downloadable from https://github.com/dww100/sct (Wright & Perkins, 2015 ▸). The new *SCT* release in the *SASSIE-web* Calculate module (Fig. 2 ▸ *b*) features an improved user interface (including the acceptance of DCD coordinate files) and modules which facilitate its use in modelling workflows (*e.g.* comparing the theoretical and experimental curves).

### 2.7. Scattering curve analyses   

The final stage of the modelling (Fig. 2 ▸ *b*) is of course the comparison of the simulated scattering profiles from the structural coordinates with the experimental SAXS and/or SANS scattering curves in order to identify the best-fit structures. In the Analyze module of *SASSIE*, the χ^2^ filter module offers one approach. This module compares the theoretical scattering profiles with the interpolated experimental data. The user supplies an input experimental data file containing three columns – *Q*, *I*(*Q*) and error of *I*(*Q*) at each *Q* value – together with the *I*(*0*) value from the Guinier *R*_G_ analysis or the *P*(*r*) analysis. In the output, three mathematical options are provided to evaluate the quality of the comparison, namely the reduced χ^2^, χ^2^ and the Pearson χ^2^. To process the simulated scattering curves produced by *SCT*, the module that was originally termed *SCTPL* (Perkins *et al.*, 2008 ▸, 2011 ▸) is now renamed as *SCT Analyze* to clarify its purpose. As with the χ^2^ filter module the user is given a choice of comparison metric (*R* factor or reduced χ^2^). Typical *R* factors for best-fit SAXS or SANS analyses are between 2 and 8%, and typical best-fit χ^2^ values are around 1–2 (Table 1 ▸).

The Analyze module also helps deduce the biological significance of the final best-fit structures (Fig. 2 ▸ *b*). Starting from the DCD frames file or the PDB coordinate files, the density plot module generates files (using the Gaussian cube file format) with volumetric data. Often, this is used to visualize sub-ensembles of structures that give the best agreement with experimental data, in addition to views of all the trial structures generated. The resulting envelopes can be visualized in molecular viewing packages such as *VMD*. Given the atomistic nature of the best-fit structures, further analyses of these best-fit structures become possible; *e.g.* these may include the calculation of electrostatic surface charge effects.

### 2.8. User support and community   

The CCP-SAS web site with background, reports and publications is at http://www.ccpsas.org/. At its inception, CCP-SAS benefitted from resources from the NIST Center for Neutron Research in the USA, and the Diamond Light Source and the ISIS Pulsed Neutron Source in the UK. But, being a CCP, CCP-SAS aims to create a community of users and provide a training infrastructure, in addition to developing software that suits the need of experimentalists. Its outreach strategy involves regular meetings, tutorials and workshops with users at scattering facilities, and engaging wider audiences at conferences through targeted training activities. In terms of maintenance, most users of CCP-SAS software will not have direct access to support staff. Consequently, detailed yet comprehensible documentation is required. Each completed package or module developed within CCP-SAS has its own online documentation. The documentation outlines the elements of each module and its interface and how to use it. In addition, worked examples are provided with accompanying files based on previous successful projects. This documentation is provided online at https://sassie-web.chem.utk.edu/sassie2/docs/ *via* a prominent Docs tab on the home page (Fig. 3 ▸ *a*). No matter how well documented and tested the software is, there will always be new user issues. A CCP-SAS Google Group provides user support (https://groups.google.com/forum/#!forum/madscatt). This is linked to the *SASSIE-web* interface so that users can directly report issues in specific modules, raise new features to be added and propose the best ways to tackle new projects. *CCP-SAS* is also actively engaged in supporting the larger SAS software developer community.

## 3. Results: applications of *SASSIE* atomistic modelling   

**Figure 4:**
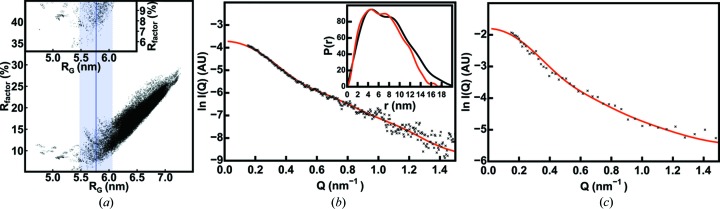
The *SASSIE* modelling workflow for monomeric human IgA1 (Hui *et al.*, 2015 ▸). This work and that in Fig. 5 ▸ was presented at the 16th International Conference on Small-Angle Scattering at the Technische Universität Berlin, Germany, on 13–18 September 2015. (*a*) The goodness-of-fit *R* factors for the calculated *I*(*Q*) curves from 146 484 hydrated IgA1 structures were calculated relative to the *I*(*Q*) curve extrapolated to zero concentration. The *R* factors were plotted against the *R*_G_ value calculated for each hydrated model. The experimental *R*_G_ value of 5.77 ± 0.04 nm (unless otherwise stated, uncertainties are reported as one standard deviation) is shown by the vertical blue line, and a coloured band indicates the ±10% range of X-ray *R*_G_ values used for filtering the best-fit models. The inset shows an expanded view for the *R* factors below 10%. (*b*) The SAXS curve fit for the median best-fit structure for full-length IgA1 identified from a cluster of 112 best-fit structures (Figs. 1 and 5). The calculated *I*(*Q*) and *P*(*r*) curves are shown in red and compared with the experimental data in black. (*c*) The SANS fit for the unhydrated structure corresponding to the best-fit SAXS hydrated structure is also shown.

**Figure 5:**
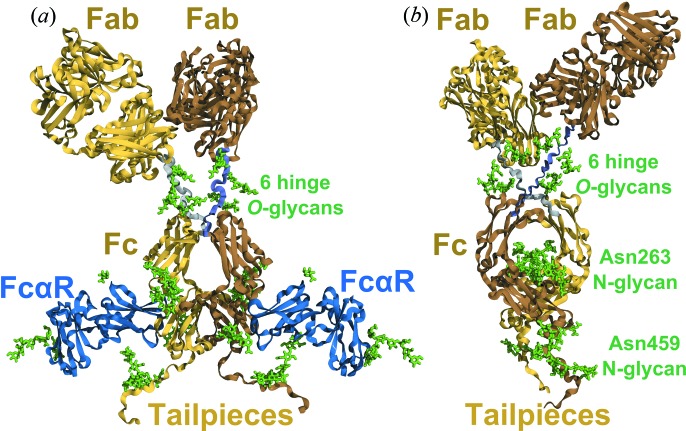
Final molecular modelling results for human IgA1 antibody (Hui *et al.*, 2015 ▸). The protein main chain is shown as a yellow ribbon. The structure was taken from the median of the 112 models in the best-fit cluster. The *O*-glycans at Thr225, Thr228 and Ser232 in the hinge and the *N*-glycans at Asn263 and Asn459 in the Fc region and tailpiece, respectively, are shown as green sticks. (*a*) View face on to the Fc region in the best-fit Y-shaped IgA1 structure. The two FcαR sites on the Fc region are shown occupied by two FcαR receptors (blue: PDB code 1ow0; Herr *et al.*, 2003 ▸). (*b*) View edge on to the Fc region in this best-fit IgA1 structure. This view was rotated by 90° about a vertical axis, and the two blue FcαR receptors were deleted. This view shows the location of the *O*-glycans and *N*-glycans in IgA1 as green sticks.

The first six examples of *SASSIE*-driven atomistic scattering modelling below illustrate the breadth of its applications in structural biology and how biologically useful information is obtained. Both single-chain proteins and multimers have been analysed in a range of *SASSIE* workflows (Table 1 ▸), but all driven from the same user interface. Common to all six biology projects (Fig. 1 ▸ and Table 1 ▸) is the definition of a correctly formatted initial structure (including the addition of hydrogen atoms), which is energy minimized with *NAMD* or *CHARMM* using the *CHARMM27* or *CHARMM36* force field (MacKerell *et al.*, 1998 ▸). This initial structure is then subjected to MC simulation to generate an ensemble of physically relevant structures. Theoretical scattering profiles of each structure in the ensemble are compared with the experimental SAXS and/or SANS data to define the best-fit structures, from which new biological insight is obtained, as exemplified in Figs. 4 and 5 (§3.5). The resulting ensembles can then form the basis of further studies. The seventh example illustrates an application of *SASSIE* to a synthetic polymer system.

### 3.1. Solution structure of a three-domain protein Gag   

The first system to be studied by *SASSIE* was HIV-1 Gag, a long polypeptide chain which consists of four domains (MA, CA, p2 and NC) connected to four long flexible linkers. The human immunodeficiency virus type 1 (HIV-1) Gag polyprotein leads to the efficient assembly of virus-like particles in mammalian cells after this is cleaved by a viral protease. Gag is composed of three well defined immature proteins, matrix (MA), capsid (CA) and nucleocapsid (NC), alongside p2 whose structure is not well known (Fig. 1 ▸). These form virus-like particles when exposed to nucleic acids and their assembly will be governed by the solution structure of Gag.

Because molecular structures for the MA, CA and NC domains have been determined by crystallography and NMR, the solution structure of monomeric Gag could be modelled from SANS data and *SASSIE* modelling (Datta *et al.*, 2007 ▸). The primary unknown was the long flexible linkers that join these three domains, including flexible linkers within both the CA and NC domains. The Monomer MC module was used to generate eight groups of 600 structures (totalling 4800) by varying specified main chain φ and ψ angles in five peptide linkers (marked with arrows in Fig. 1 ▸). This large ensemble of trial structures was output into a DCD file, and then these were energy minimized using *CHARMM*. The resulting scattering analysis of the 4800 models gave χ^2^ values that ranged between 1.2 for four groups of best-fit structures to as high as 30 for the other 16 groups of structures. While no single correct structure was identified, the key result from the best-fit ensembles showed that unbound Gag was folded over into a compact shape with the N-terminal MA and C-terminal NC domains close to each other, *i.e.* such a structure undergoes a conformational change when this is assembled into a virus. This modelling workflow is described in more detail in the online tutorial for *SASSIE*.

In a recent similar study, the examination of the bacterial single-stranded DNA binding protein from SAXS and SANS data showed that the protein’s long disordered C-terminal tails were relatively collapsed around the well defined N-terminal core protein structure that binds single-stranded DNA, and compacted further upon binding single-stranded DNA, which was at variance with the previously hypothesized model (Green *et al.*, 2016 ▸). To visualize this outcome by atomistic modelling, *SASSIE* was first used to generate the most compact atomistic structure possible for the core and tails. Next, 10 000 structures for the full-length protein were generated from MD, in which the tails were allowed to adopt all stereochemically permitted disordered conformations. Careful allowance for hydration was required for the SAXS fits; however, the SANS data could be directly compared with the unhydrated models because the surface hydration shell of bound water molecules does not contribute to the SANS data. Finally, the resulting curve fits showed that the most collapsed ensemble of tail structures best represented the experimental scattering curves.

### 3.2. Solution structure of a two-domain protein Ub_2_   

Polyubiquitination is a post-translational modification of an intracellular protein by an ubiquitin dimer that signals major cellular events. Different signalling pathways result, depending on the isopeptide linkages formed between the C-terminus of the so-termed ‘distal’ ubiquitin (Ub) and the ∊-amine of any one of seven lysine residues on the other ‘proximal’ Ub. For example, Ub dimers linked by Lys48 or Lys63 mediate proteosomal degradation and DNA repair, respectively. The Ub dimer formed through Lys27, termed K27-Ub_2_ (Fig. 1 ▸), has unique biochemical properties (Castañeda, Chaturvedi *et al.*, 2016 ▸; Castañeda, Dixon *et al.*, 2016 ▸).

The ubiquitin dimer is joined by an isopeptide bond between two monomers. Of interest is that the structure and dynamics of K27-Ub_2_ were examined jointly by NMR structural constraints, SANS data and ensemble modelling by *SASSIE* (Castañeda, Dixon *et al.*, 2016 ▸). The sparse ensemble selection method was used to determine representative conformational ensembles for K27-Ub_2_ for the NMR analyses. The ensembles of 23 000 sterically allowed structures were generated using the monomer MC routine in *SASSIE* to vary residues 72–76 of the distal Ub monomer that were connected to Lys27 in the proximal Ub monomer (arrow in Fig. 1 ▸). Residues 72–76 were considered to be flexible. Importantly, the fit for the residual dipolar couplings from NMR was significantly improved if two K27-Ub_2_ conformers and not one were considered. Independent *SASSIE* fits of the experimental SANS data using these 23 000 structures showed that a one-conformer ensemble gave good agreement, and two-conformer ensembles slightly improved the agreement. Similarity with the structure of the ligand-bound state of the Ub dimer linked *via* Lys48 suggested a possible receptor for K27-Ub_2_, which was then confirmed experimentally. The biological importance of this dimer was revealed by studying the interaction between K27-Ub_2_ and its receptor by molecular docking based on NMR signal perturbation and paramagnetic spin labelling. In this receptor complex, surface-exposed hydrophobic patches on each of the two Ub proteins formed a V-shaped groove in the dimer that interacted specifically with the receptor inside its V, thus rationalizing the distinct biochemistry of this particular Ub dimer.

### 3.3. Solution structure of a six-domain protein MASP   

The lectin pathway of the complement system in plasma is activated by complexes on pathogen surfaces that comprise a recognition component (MBL: mannose-binding lectin) that binds multivalently to mannose residues on the pathogen. MBL forms complexes with an MBL-associated serine protease (MASP) that leads to MASP activation in a manner that is unclear. MASP exists as a homodimer, with two six-domain monomers tightly bound in an antiparallel arrangement by their N-terminal domains (Fig. 1 ▸).

The dimeric MASP proteins are composed of two copies of six protein domains joined by short linkers; the dimer is formed by the tight noncovalent pairing of the N-terminal CUB–EGF domain pair (Fig. 1 ▸). The solution structure of the full MASP dimer with 12 domains was not known, and unravelling this structure was critical to understanding MASP activation (Nan *et al.*, unpublished work). The *SASSIE* modelling of SAXS data for MASP was performed alongside crystallographic investigation of the same proteins and was achievable despite the large size of this protein. An initial linear six-domain monomer model was built using a combination of existing domain structures and new crystal structures for the three N-terminal domains that form the dimer. When full-length dimeric MASP was studied by analytical ultracentrifugation, the experimental sedimentation coefficients *s*_20,w_ of 5.4–5.9 S were notably larger than those of 5.3–5.5 S calculated from the initial linear model. From the X-ray *R*_G_ analyses, the experimental values of 7.5–7.9 nm for full-length MASP were likewise notably smaller than those of 8.2–9.0 nm calculated from the initial linear model. Both differences indicated that the solution structures of MASP were more bent than the initial linear model.

In the *SASSIE* workflow, the Monomer MC module was used to sample MASP configurations by varying one or four inter-domain linkers in the three- and six-domain proteins (arrows in Fig. 1 ▸). As many as 30 910 trial conformations (Table 1 ▸) were generated using maximum rotation steps for the peptide φ and ψ angles of up to 80°. The *SCT* scattering curves were calculated from coarse-grained sphere models using a cutoff of four atoms per sphere in a grid with a cube side of 0.530 nm. A hydration sphere shell corresponding to 0.3 g of water per gram was added to each unhydrated model. Three N-linked glycan chains are present in MASP. These could not be considered during the *SASSIE* modelling, which was performed without explicit glycans added. Once the best-fit MASP structures had been identified, glycans in extended conformations were added to these, whereupon the *R* factors were improved to reduced final values of 4.6–5.2% (Table 1 ▸). The *SASSIE* modelling showed that much improved curve fits resulted from bent full-length atomistic MASP structures, compared to the extended initial structure. This key result revealed that MASP existed as flexible structures in which the two SP domains at the tips of the MASP dimer were able to move towards each other. Although this hypothesis is not proven, the modelling suggests that the MASP domains are flexible and that the two SP domains at the tips of the MASP dimer may come sufficiently close to explain how MASP auto-activation may take place. The MASP example, being constrained by crystal structures, showed that as many as eight variable linkers can be analysed using *SASSIE*. The incorporation of greater numbers of variable linkers requires other approaches, such as that for intrinsically disordered proteins discussed above (Green *et al.*, 2016 ▸).

### 3.4. Solution structures of IgG2 antibodies   

IgG antibodies are central to the adaptive immune response against pathogens. As therapeutics, over 300 IgG monoclonal antibodies have been approved for clinical use. The four human IgG subclasses IgG1–IgG4 in serum differ primarily in their hinges, where their lengths are 15, 12, 62 and 12 amino acids, respectively. Atomistic antibody modelling by *SASSIE* is an ideal method to investigate how the Fab regions are connected to the Fc regions through two long flexible linkers (or hinges) (Fig. 1 ▸). Because the two Fab and Fc regions are largely independent of each other, antibody modelling is distinct from the examples of the linear two- to six-domain proteins above.

*SASSIE* was used to study the structure of a monoclonal human IgG2 antibody that was characterized by SANS (Clark *et al.*, 2013 ▸). The atomistic modelling was initiated by a homology model for human IgG2 that was generated from a crystal structure for mouse IgG2a. This homology model was subjected to MC simulation by sampling three residues in the upper hinge in random rotational steps of up to 10°. Each of the resulting 56 511 conformations was subjected to energy minimization, followed by a generalized Born-implicit solvent MD simulation, and another round of energy minimization. From comparison between the experimental and calculated SANS curves, the standard plot of χ^2^ *versus R*_G_ values showed a U-shaped distribution in which the best-fit structures appeared at the minimum (*e.g.* Fig. 4*a* below). Visual inspection showed that the conformational space about the hinge had been well sampled, but only a small set of conformations were in agreement with experiment with χ^2^ < 2. An asymmetric arrangement of the two Fab regions compared to the Fc region was identified. This ensemble of structures was consistent with the scattering data; however the configurations may or may not be energetically plausible. To complete this study, energetic information from simulations was used to refine the ensemble of best-fit structures for IgG2. The widely used Adaptive Poisson Boltzmann Solver implemented in *SASSIE-web* was used to calculate solvation free energies from the ensemble models (Baker *et al.*, 2001 ▸; Dolinsky *et al.*, 2004 ▸, 2007 ▸). These solvation energies acted as a further filter on acceptable models and produced a reduced subset of structures exhibiting lower free energies. The use of free-energy constraints meant that the final models corresponded to more physically reasonable structures. This software technology was able to identify specific interactions known to affect function and/or chemical stability, and illustrated a new approach made possible because of the modules in *SASSIE*.

In related studies that used the older *SCT* and *SCTPL* approach, atomistic analyses for monoclonal human IgG1 and IgG4 were based on known crystal structures for the Fab and Fc regions. MD simulations were used to vary the hinge conformations in order to interpret the SAXS and SANS data (Rayner *et al.*, 2014 ▸, 2015 ▸). The outcome also revealed asymmetric IgG structures. The resulting atomistic structures explained why human IgG1 binds to its receptors and complement more readily than human IgG4.

### 3.5. Solution structures of IgA1 antibodies   

IgA1 and IgA2 antibodies are important in mucosal immunity. IgA nephropathy is a leading cause of chronic kidney disease, in which the deposition of IgA1-containing immune complexes in the kidney often leads to renal failure. The structure of IgA1 is unusual in possessing two long 23-residue hinges between the Fab and Fc regions. These hinges are *O*-glycosylated with GalNAc.Gal.NeuNAc moieties, and these *O*-glycans are often found at reduced levels in patients with IgA nephropathy.

The *SASSIE* modelling of SAXS and SANS data (Fig. 4 ▸) investigated the impact of glycosylation on the IgA1 solution structure. To clarify whether variations in these *O*-glycans affect IgA1 function and disease, human IgA1 was studied with four different *O*-glycosylation levels (Hui *et al.*, 2015 ▸). Analytical ultracentrifugation showed that all four IgA1 samples were monomeric with similar sedimentation coefficients *s* ^0^_20,w_. SAXS and SANS data in light and heavy water, respectively, for the four IgA1 samples revealed no conformational changes between the four IgA1 samples. Interestingly, the SANS data acquired in heavy water suggested that a reduction in *O*-glycan content was correlated with an increase in the propensity for IgA1 to aggregate, *i.e.* this may be related to the onset of IgA nephropathy. The *SASSIE* modelling workflow for IgA1 proceeded in two stages. First, a truncated IgA1 structure was modelled from crystal structures for each of the human IgA1 Fab and Fc regions, with the hinge and C-terminal regions modelled *de novo*. Two *N*-glycan chains at Asn263 in the Fc region in the crystal structure were also varied. This IgA1 structural model was energy minimized (Fig. 5 ▸ *b*). MC simulations of the hinge conformations resulted in 172 833 trial models whose calculated scattering curves were compared to the SANS data to identify the best-fit truncated IgA1 models. Second, these best-fit truncated IgA1 structures were completed by adding structures for the two *N*-glycosyl­ated C-terminal tailpieces obtained from MD simulations to give 146 484 trial models for full-length IgA1 (Fig. 4*a* ▸). Principal component analysis identified four major IgA1 conformations. One of these conformations gave very good SAXS and SANS curve fits (Figs. 4 ▸ *b* and 4 ▸ *c*). Whilst no structural variation was found with differing glycosylation levels, in agreement with experiment, the addition of six *O*-glycans to the hinges improved the SAXS fit and resulted in final *R* factors of 4.8–6.2%. The final ensemble of 113 best-fit models showed that the solution structures of full-length IgA1 possessed extended hinges and asymmetrically positioned Fab and Fc regions. Ample space in IgA1 was revealed for the functionally important binding of two FcαR receptors to its Fc region (Fig. 5 ▸ *a*).

### 3.6. Assembly of the Hfq-mRNA complex   

The modelling of a large protein–RNA complex was achieved by the combination of *SASSIE* structural modelling with evidence from chemical footprinting. The hexameric RNA-binding protein Hfq from *Escherichia coli * (Fig. 1 ▸) enables the regulation of mRNA by bacterial small noncoding RNAs (sRNAs) in response to stress and other environmental signals. In order to determine how the Hfq hexamer brings sRNA and mRNA together in the proper orientation for this regulatory function, *SASSIE* was used to model SAXS data for unbound Hfq (6 × 102 residues) and mRNA (284 nucleotides) and their complex (Peng *et al.*, 2014 ▸). The *SASSIE* modelling was based on the crystal structure of the core hexameric Hfq complexed with two small RNA heptamers (Fig. 1 ▸; Wang *et al.*, 2013 ▸).

Kratky plots of the SAXS data showed that free mRNA has an extended structure, while Hfq has a compact globular structure. When Hfq was added to mRNA, the change in the appearance of the Kratky plot showed that the extended mRNA structure had compacted and wrapped itself around the Hfq protein (Fig. 1 ▸). To verify these scattering results, initial atomistic models were built. That for the full-length *E. coli* Hfq hexamer was created by appending its disordered N- and C-terminal residues (residues 1–5 and 66–102, respectively) to the crystal structure of the HfQ core. Those for the mRNA models (273 nucleotides) were generated from three-dimensional structures for six RNA fragments using the *MC-Sym* web server (Parisien & Major, 2008 ▸). These six RNA models were merged to give an L-shaped mRNA structure with a flexible pivot between nucleotides 128 and 129. This was energy minimized. The full Hfq–mRNA complex was formed by the superimposition of the initial structures for Hfq and mRNA with the crystal structure of the Hfq core complexed with the two small RNA heptamers. The scattering modelling of the complex was based on variations of both the Hfq and mRNA models. Thus MC simulations were performed in which the terminal residues 1–5 and 66–102 in Hfq were allowed to move, while the Hfq core was held fixed. The simulations held the mRNA structure fixed except for the pivot between nucleotides 128 and 129. From this, 917 models from the 19 132 generated for the complex were accepted to give good scattering fits after comparison with the scattering data. The key result from the 917 models showed that the full-sized mRNA structure could bend around both sides of the Hfq hexamer (Fig. 1 ▸). This outcome from the *SASSIE* modelling provided evidence for how Hfq–mRNA binding could specifically distort mRNA such that sRNA could bind to exposed regions of mRNA, thus explaining the translational control achieved by the sRNAs.

### 3.7. The structure of ‘bottlebrush’ polymers   

Bottlebrush polymers are a technologically interesting class of macromolecules. As the name suggests, multiple side chain grafts radiate from the polymer backbone, impacting chain flexibility, interactions, self-assembly and dynamics (Zhang *et al.*, 2014 ▸). Unlike the biological examples above, for which the starting coordinates were generated from crystal structures, this study used the *AMBER* MD package (Case *et al.*, 2016 ▸) to create a norbornenyl end-functionalized poly(lactide) macro­monomer (NB-PLA) with a poly NB (PNB) backbone and PLA side chains. This monomer was replicated 25 times. The resulting polymer was then solvated in tetrahydrofuran, energy minimized in a periodic box and brought to equilibrium. The largest simulated system comprised PNB_25_–*g*-PLA_19_ and 34 298 tetrahydrofuran molecules. Trajectories were output every 100 ps and used to compute the scattering curve, which could then be compared with experimental SANS data. *SASSIE* was used to automate the processing of many simulation frames and to filter for those conformations whose statistically averaged structures showed better agreement. This analysis demonstrated that structures with an *R*_G_ value of ∼3.7 nm gave better fits to the SANS data. Moreover, the best-fit trajectories also suggested that the scattering form factor could be well approximated by a short rigid cylinder or ellipsoid of revolution.

## 4. Discussion: the outlook for *SASSIE-web*   

SAXS and SANS experiments are powerful experimental methods for elucidating the solution structures of biological macromolecules at low structural resolution. The future development of SAXS and SANS will require the inclusion of advanced atomistic modelling to analyse scattering data properly. There are three main advances offered by CCP-SAS: (i) providing an open-source software environment for developers that (ii) facilitates the easy uptake by users of advanced molecular modelling for the interpretation of SAXS and SANS experiments, and (iii) is seamlessly linked to high-performance computing resources offered through the *SASSIE-web* front end. *SASSIE-web* provides a unified workflow framework to molecular simulation engines for both MC and MD, scattering calculators (*SasCalc* and *SCT*), and other structure building, job management and general analysis modules (Fig. 2 ▸ *b*). The foundation of this platform is the *GenApp* deployment infrastructure, developed within the CCP-SAS project, which enables the generation of web and standalone GUI applications from the underlying code and provides interfaces to high performance computing resources (Fig. 2 ▸ *a*). The *SASSIE* applications summarized above illustrate how advanced computational modelling will assist scattering projects in addition to the traditional experimental SAXS and SANS data analyses. While daunting at first sight, it is an important challenge for *SASSIE* to make the atomistic modelling as easy as possible.

The uptake of CCP-SAS software is increasing, with over 200 users registered on the *SASSIE-web* server to date. At the SAS2015 Conference in Berlin, about 24 protein, nine protein–lipid, eight protein–DNA and two chemical physics projects were reported to be under way. Meanwhile, the number of third-party web applications such as *WillItFit* is growing, with five groups in various stages of using *GenApp* and CCP-SAS compute resources to deploy their code. Further enhancements for *SASSIE* will include additional modules to make the modelling workflow easier for the user. For example a new module, termed *PDB-Rx*, is in preparation for the Build set of *SASSIE* modules to help rectify errors identified in *PDB-scan* or in the user-supplied PDB coordinate file, or to complete any omissions (Wright *et al.*, 2016 ▸). The goal of *PDB-Rx* will be to automate not only the ‘tidying’ and completion of PDB files, but also the preparation of structures using the *CHARMM* force field for use in the *SASSIE* simulations. The analysis of new macromolecular systems with intrinsic disorder is becoming increasingly important, following the recognition that many human proteins show disorder. For this, it becomes necessary to develop models that represent ensembles of disordered structures, which is what *SASSIE* does well (Datta *et al.*, 2007 ▸; Green *et al.*, 2016 ▸). As illustrated by the MASP and IgA1 examples above, new modules will also be needed to incorporate glycan chains more easily into trial structures, rather than adding the glycans after the best-fit structure is determined. It is a limitation of standard molecular modelling software that the non-protein or non-nucleic acid elements of many systems are not included in many biologically focused packages. And as solution-derived structures become even more commonplace there will also be a need to revisit the deposition of best-fit atomistic structures in their own right in public databases (Wright & Perkins, 2015 ▸), together with the experimental data used to derive these structures.

Modules that are specific for soft condensed matter systems important in physical chemistry (Higgins & Benoit, 1996 ▸; Gabrys, 2000 ▸) have not been described in this article either. However, the development of atomistic models for polymers, surfactant micelles and lipid nanodiscs appropriate for SAS modelling is in progress within CCP-SAS. Besides the usual difficulty in generating a representative starting structure, these systems suffer from a lack of good appropriate force fields. For soft matter systems, it will also be necessary to account for concentration-dependent inter-particle effects, unlike the case of biological systems where scattering data are often extrapolated to infinite dilution. Models of soft matter systems will need to be large enough to allow several micelles to form, and to allow for models showing a realistic degree of polydispersity (not required in biological systems) to be generated. Coarse graining will be essential to achieve mol­ecular models of such systems, particularly when large-scale movements of molecules (rather than just torsional or bending motions within one molecule) are required to generate potential structures for comparison with scattering patterns. This work will also be driven by the desire to model more complex mixed systems, such as surfactant micelles with polymers or colloidal particles, which are the focus of typical standard soft matter small-angle scattering studies.

An exciting prospect going forward is the development of ever more robust, easy to use tools that will eventually enable the SAS user community to routinely take full advantage of combining rapid SAXS and SANS atomistic modelling with data from complementary disciplines such as analytical ultracentrifugation, X-ray crystallography and NMR spectroscopy (§§2.2–2.4 [Sec sec2.4]), as well as electron microscopy. Indeed the combination of different experimental methods provides new insights not available from one method alone, as demonstrated by the *SASSIE* applications (Table 1 ▸). In addition, the availability of experimentally founded atomistic models allows us to make use of the many programs available in the molecular modelling community for statistical analyses, the evaluation of energetics and the calculation of parameters relevant for other structural techniques such as NMR.

One benefit of being part of the UK’s CCP system, which provides an effective means of focusing computational resources for selected communities, is the opportunity to interact regularly with other CCP groups, many of which have overlapping and intersecting interests. The best-known of these is CCP4 (crystallography) (Winn *et al.*, 2011 ▸). As well as CCP4, CCP-SAS overlaps with CCP-EM (electron cryo­micro­scopy) (Wood *et al.*, 2015 ▸), CCPN (macromolecular NMR spectroscopy), CCP5 (simulation of soft condensed phases) and CCPBioSim (biomolecular simulation) (Lonsdale *et al.*, 2014 ▸). CCP-SAS provides an ideal path forward, not only to tackle the advancement of co-refinement of data from various techniques, but also to advance the soft matter agenda. Although CCP-SAS was initially funded as a joint US/UK venture, the CCP-SAS consortium viewed the project from the outset as more global. As such the project is actively seeking collaborations with and engagement by the global SAS community and welcomes inquiries into creating joint efforts for the benefit of that community.

## Figures and Tables

**Table 1:** Atomistic modelling projects completed using *SASSIE*

Biological system	HIV-1 Gag	Ubiquitin dimer (Ub_2_)	MASP dimers	Human IgG2	Human IgA1	Hfq-mRNA
Experimental data	Neutron scattering (NG3 30 m and NG7 30 m at NIST)	600 and 800 MHz NMR structures; neutron scattering (NG3 30 m at NIST)	X-ray crystallography; analytical ultracentrifugation; X-ray scattering (BM29 at ESRF)	Neutron scattering (NG3 30 m and NG7 30 m at NIST)	Analytical ultracentrifugation; X-ray scattering (ID02 at ESRF); neutron scattering (SANS2d at ISIS)	X-ray scattering (12-ID-B at APS); chemical footprinting
Starting models for *SASSIE*	NMR structures for MA and NC; crystal structure for CA	NMR structure for the Ub monomer	3 crystal structures for CUB1–EGF–CUB1, CUB1–SCR1 and SCR1–SCR2–SP	Crystal structure for full-length mouse IgG2a	Crystal structures for the IgA1 Fab and Fc regions	Crystal structure for the core Hfq–mRNA complex
Structurally varied linker(s) in *SASSIE*	5 flexible linkers between the MA, CA and NC domains	C-terminal residues 72–76 of the distal Ub in the Ub dimer	2 linkers in CUB1–EGF–CUB1; 5 linkers in full-length MASP	3 amino acids in the IgG2 upper hinge	2 *O*-glycosyl­ated hinges; 2 N-glycans; 2 N-glycosylated tailpieces	Residues 1–5 and 66–102 in the Hfq hexamer; the 128/129 hinge in mRNA
Number of models used in *SASSIE*	4800 HIV-1 Gag models	30 000 K27-Ub_2_ dimer models	1982–4517 models for CUB1–EGF–CUB1; 6173–30 910 models for full-length MASP	56 511 IgG2 models	172 833 truncated IgA1 models; 146 484 full-length IgA1 models	24 991 Hfq models; 27 427 mRNA models; 19 132 models for the complex
Molecular mass (kDa)	53	17 (dimer)	75 and 170	150	164	67, 96 and 163
Experimental *R*_G_ value (nm)	3.4	18.5–19.4 for the K27-Ub_2_ dimer	3.79–3.87 for CUB1–EGF–CUB1; 7.54–7.93 for full-length MASP	4.75	5.93	3.36 nm (Hfq); 6.81 nm (mRNA); 5.80 nm (complex)
*Q*-range† of scattering curve (nm^−1^)	0.09–2.50 (neutrons)	0.30–4.0 (neutrons)	0.06–2.20 (X-rays)	0.07–3.00 (neutrons)	0.13–2.10 (X-rays); 0.18–1.6 (neutrons)	0.05–10.07 (X-rays)
Final *R* factor or χ^2^ value	1160 HIV-1 Gag models with χ^2^ of 1-2	χ^2^ of 1.02 – 2.36 for 5 dimer conformational clusters	*R* factor of 4.1–4.2% for CUB1–EGF–CUB1; 4.6–5.2% for full-length MASP	1160 IgG2 models with χ^2^ < 2	*R* factor for full-length IgA1: 6.1–6.4% (X-rays); 8.7–11.3% (neutrons)	917 Hfq–mRNA models with χ^2^ < 1.5
Reference	Datta *et al.* (2007 ▸)	Castañeda, Chaturvedi *et al.* (2016 ▸), Castañeda, Dixon *et al.* (2016 ▸)	Nan *et al.* (2017 ▸)	Clark *et al.* (2013 ▸)	Hui *et al.* (2015 ▸)	Peng *et al.* (2014 ▸)

† *Q* is defined as 4πsinθ/λ, where 2θ is the scattering angle and λ is the wavelength.
